# Construction and Mechanism of Janus Nano-Graphite Reinforced Foam Gel System for Plugging Steam in Heavy Oil Reservoirs

**DOI:** 10.3390/gels10110721

**Published:** 2024-11-07

**Authors:** Zhongzheng Xu, Yuxin Xie, Xiaolong Wang, Ning Sun, Ziteng Yang, Xin Li, Jia Chen, Yunbo Dong, Herui Fan, Mingwei Zhao

**Affiliations:** 1 State Key Laboratory of Deep Oil and Gas, China University of Petroleum (East China), Qingdao 266580, China; upc_xzz@163.com (Z.X.); m18391223655@163.com (Y.X.); wangxlupc@126.com (X.W.); 19863704412@163.com (Z.Y.); lixindesuzi@163.com (X.L.); chenjia9248@163.com (J.C.); 15590262179@163.com (Y.D.); herui8503@163.com (H.F.); 2 Key Laboratory of Unconventional Oil & Gas Development, China University of Petroleum (East China), Ministry of Education, Qingdao 266580, China; 3 Key Laboratory of Enhanced Oil & Gas Recovery, Northeast Petroleum University, Ministry of Education, Daqing 163318, China; dylan199161@163.com

**Keywords:** heavy oil recovery, steam-channeling control, foam stability, Janus nano-graphite stabilizer

## Abstract

High-temperature steam injection is a primary method for viscosity reduction and recovery in heavy oil reservoirs. However, due to the high mobility of steam, channeling often occurs within the reservoir, leading to reduced thermal efficiency and challenges in enhancing oil production. Foam fluids, with their dual advantages of selective plugging and efficient oil displacement, are widely used in steam-injection heavy oil recovery. Nonetheless, conventional foams tend to destabilize under high-temperature conditions, resulting in poor stability and suboptimal plugging performance, which hampers the efficient development of heavy oil resources. To address these technical challenges, this study introduces a foam system reinforced with Janus nano-graphite, a high-temperature stabilizer characterized by its small particle size and thermal resistance. The foaming agents used in the system are sodium α-olefin sulfonate (AOS), an anionic surfactant, and octadecyl hydroxylpropyl sulfobetaine (OHSB), a zwitterionic surfactant. Under conditions of 250 °C and 5 MPa, the foam system achieved a half-life of 47.8 min, 3.4 times longer than conventional foams. Janus nano-graphite forms a multidimensional network structure in the liquid phase, increasing internal friction and enhancing shear viscosity by 1.2 to 1.8 times that of conventional foams. Furthermore, the foam gel system demonstrated effective steam-channeling control in heterogeneous heavy oil reservoirs, particularly in reservoirs with permeability differentials ranging from 3 to 9. These findings suggest that the Janus nano-graphite reinforced foam system holds significant potential for steam-channeling mitigation in heavy oil reservoirs.

## 1. Introduction

Heavy oil, as an unconventional oil and gas resource, has been driving advancements in the petroleum industry [1]. However, its high viscosity, complex chemical structure, and significant proportion of heavy components have resulted in increased difficulty in its development [2,3,4]. The most commonly used methods to enhance heavy oil recovery are steam injection technologies, including cyclic steam stimulation (CSS), steam flooding, and steam-assisted gravity drainage (SAGD). These methods effectively reduce viscosity and have been widely implemented worldwide [5,6]. As production time advances, disparities in density and viscosity, along with the loosening of reservoir cementation, can lead to significant issues in thermal recovery processes, such as steam override, steam coning, and steam channeling [7]. This significantly affects thermal efficiency and oil recovery rates [8,9,10]. Controlling steam is a prerequisite for enhancing recovery efficiency. Particulate plugging agents, such as cement or hard mineral powders, are suspended in carrier fluids and used to seal highly permeable formations or fractures that serve as steam-channeling pathways [11,12]. These agents are widely applied in steam flooding for heavy oil recovery due to their low cost, high temperature resistance, strong sealing capability, and long-lasting effect. However, these agents suffer from poor injectivity, making it difficult for them to penetrate deep into the formation, which reduces their sealing effectiveness. Gel-based high-temperature sealing agents possess strong sealing capabilities and cause minimal damage to reservoirs, making them widely used in the near-wellbore region. However, gels face challenges such as poor injectability, difficulty in deep migration, and instability at high temperatures, which can lead to chemical degradation and dehydration, ultimately reducing their sealing effectiveness [13,14,15,16]. Foam fluids, owing to their unique advantages of selective sealing and efficient oil displacement, are widely used in steam injection for heavy oil reservoirs [17,18,19]. However, the poor foam stability at reservoir temperatures results in suboptimal plugging performance, hindering the efficient development of heavy oil resources. Therefore, there is a pressing need to develop a highly stable foam plugging system to provide new methods for enhancing heavy oil recovery.

Studies have reported that foam generated solely by surfactants under reservoir conditions is prone to rapid collapse. The addition of polymers to foam fluids can increase liquid viscosity, slowing the coalescence of foam bubbles due to gravity or capillary drainage [20,21,22], thus significantly improving foam strength and stability [23,24]. However, in steam conformance control, challenges such as polymer adsorption onto rock surfaces and degradation at high reservoir temperatures must be addressed [25]. Notably, research has shown that incorporating nanoparticles into foam systems can significantly enhance foam stability, attributed to the small size and surface properties of the nanoparticles [26,27,28,29]. These particles act as foam stabilizers, tightening the bubble structure and improving the foam’s resistance to high-temperature and high-salinity reservoir conditions, thus enhancing its plugging performance [30,31,32]. Kang et al. [33] demonstrated that adding nanoparticles to a foaming system extended the foam’s half-life to 18 min at 85 °C, an improvement of 14 min compared to using surfactants alone. Moreover, as nanoparticle concentration increased, the system’s resistance to gas channeling also improved. Sun et al. [34] explored the synergistic effect of surfactants and silica nanoparticles (SNPs) in enhancing foam stability, finding that foam formed with cationic surfactants and SNPs was more stable than that formed with anionic surfactants. Similarly, Hurtado et al. [35] used polyethylene glycol (PEG)-modified silica nanoparticles to improve foam stability, with the optimal concentration of 1 wt% increasing the foam’s half-life from 9 to 16 min at 90 °C. With the advancement of nanomaterials, Janus nanomaterials have garnered widespread attention due to their enhanced properties [36,37,38]. Janus nanoparticles, characterized by distinct chemical compositions or functionalities on each side, exhibit stronger interfacial activity and dispersion stability compared to homogeneous nanoparticles [39,40,41]. Depending on their morphology, Janus nanoparticles can be spherical, rod-shaped, or sheet-like [42]. Among these, Janus nanosheets possess both amphiphilic properties and a two-dimensional structure, which enhances foam film strength and prolongs the liquid drainage half-life, providing superior foam stabilization [43,44,45]. Research indicates that Janus nanoparticles, due to their differing wettability in various regions, can increase their adsorption free energy threefold. Additionally, their unique amphiphilic properties exhibit high interfacial activity and adsorption stability, making them promising candidates for enhancing foams at elevated temperatures [46,47,48].

In this study, custom-made Janus graphite nanosheets were used as high-temperature foam stabilizers to develop a highly stable, enhanced foam system for steam conformance control in heavy oil reservoirs. A high-temperature and high-pressure foam visualization apparatus was employed to evaluate the foam comprehensive performance under extreme conditions and analyze the synergistic effects of Janus graphite with anionic and zwitterionic surfactants. An optimal Janus graphite-enhanced foam system was established. The effects of Janus graphite on bubble shape, size, and film thickness were observed using an inverted microscope, while a rheometer was used to analyze the foam rheological properties before and after the addition of Janus graphite. Additionally, a self-assembled high-temperature, high-pressure core flooding system was used to evaluate the ability of Janus graphite-enhanced foam to control steam channeling in heterogeneous heavy oil reservoirs, providing insights for improving steam injection in heavy oilfield development.

## 2. Results and Discussion

### 2.1. Optimization of Temperature-Resistant Foam Agent Formulation

To evaluate the performance improvement of the selected foaming agent blend, the ratio and total concentration of the foaming agents were varied. Foam volume and foam half-life under different conditions were measured, and the formulation was optimized based on the maximum foam comprehensive value.

**Figure 1:**
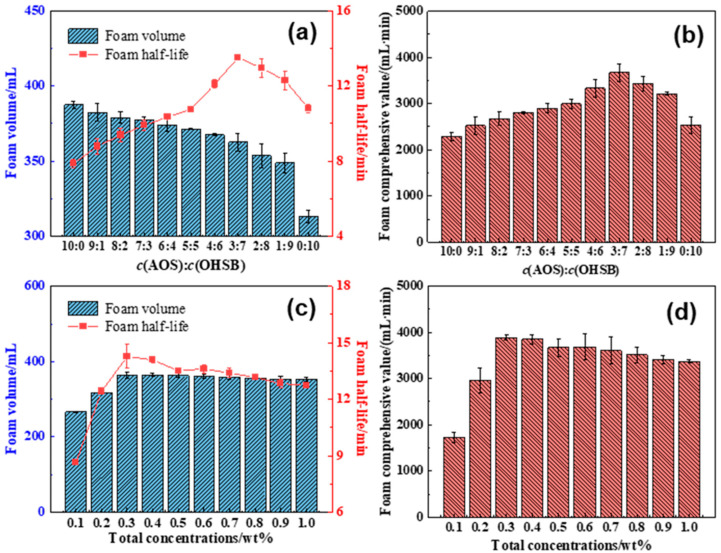
(**a**,**b**) Volume, half-life, and comprehensive value of foams with different compounding ratios of foaming agents. (**c**,**d**) Volume, half-life, and comprehensive value of foams with different total foaming agent concentration.

As shown in Figure 1a,b, at a constant total foaming agent concentration, changing the AOS/OHSB ratio resulted in significant differences in foam performance. With increasing OHSB content, foam volume decreased, while the foam half-life initially increased to a peak and then gradually declined. The adsorption of foaming agent molecules at the gas–liquid interface affects the interfacial film strength, which in turn influences foam stability. Electrostatic interactions between the negatively charged AOS groups and the positively charged OHSB groups lead to a more compact arrangement of molecules at the interface within an optimal ratio, increasing both film thickness and strength, thus extending foam half-life. Based on the maximum foam comprehensive value, the optimal ratio of c(AOS):(OHSB) = 3:7 was selected. At this ratio, the foam volume reached 362.6 mL, the foam half-life was 13.5 min, and the foam comprehensive value was 3675.1 mL·min.

As shown in Figure 1c,d, when the foaming agent ratio was fixed at c(AOS):(OHSB) = 3:7, the foam performance varied with the total foaming agent concentration. Both the foam volume and foam half-life initially increased and then decreased as the total foaming agent concentration increased. When the concentration exceeded a certain threshold, the adsorption of the two foaming agents at the gas–liquid interface reached saturation, leading to micelle formation within the solution. This affected the interface film properties, causing a slight decrease in both foam volume and foam half-life. The optimal total foaming agent concentration, based on the maximum foam comprehensive value, was found to be 0.3 wt%, where the foam volume was 363.4 mL, the foam half-life was 14.3 min, and the foam comprehensive value reached 3890.1 mL·min.

### 2.2. Evaluation of Molecular Interactions in High-Temperature Foaming Agents

**Figure 2:**
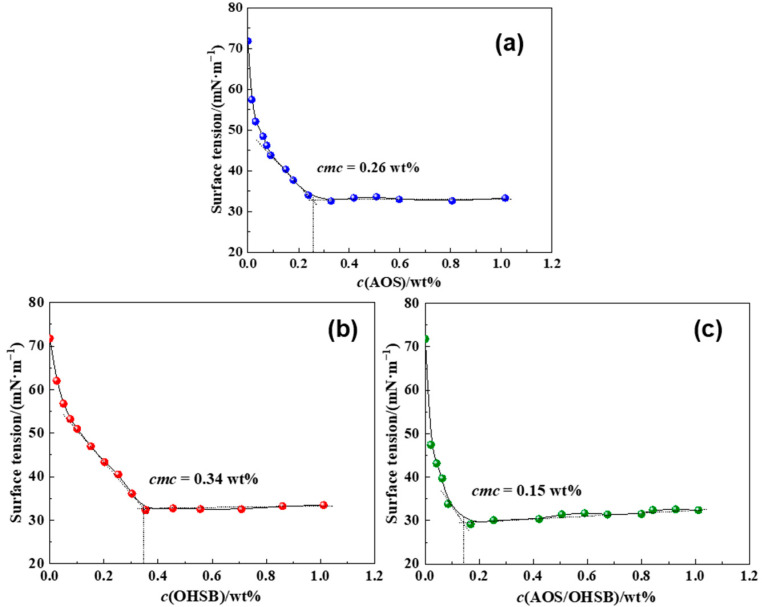
Surface tension changes with concentration of different foaming agent solutions. (**a**) AOS. (**b**) OHSB. (**c**) AOS/OHSB.

To further investigate the molecular interactions between the two combined foaming agents, the interaction parameter β was used to analyze the nature and strength of the interactions, and to determine whether the agents exhibit a synergistic effect at the gas–liquid interface and within the solution. The absolute value of the interaction parameter β indicates the strength of the molecular interactions. When β < 0, attractive forces between the two foaming agents exist. And if there is $\left|\beta \right|>\left|\ln(c_{1}/c_{2})\right|$ at the same time, it suggests a strong synergistic effect. If β > 0, repulsive forces are present. When β ≈ 0, the two foaming agents mix ideally, with negligible molecular interactions. The molecular interaction parameter β is calculated based on the critical micelle concentration (CMC) of different foaming agent solutions. As shown in Figure 2, using surface tension measurements, the CMC values of AOS, OHSB, and their combination were determined to be 0.26 wt% (0.009 mol·L^−1^), 0.34 wt% (0.010 mol·L^−1^), and 0.15 wt% (0.005 mol·L^−1^), respectively. The CMC of the combined foaming agent solution is lower than that of the individual agents, indicating a closer arrangement of molecules at the gas–liquid interface and suggesting a synergistic effect that enhances the overall foam performance. As shown in Table 1, the inter-molecular interaction parameter β between AOS and OHSB is negative, and its absolute value is greater than ln(*c_1_*/*c_2_*), indicating a strong attractive interaction between the two surfactant molecules. Therefore, the synergistic enhancement effect resulting from the attraction between AOS and OHSB molecules further improves the overall foam performance when these surfactants are combined.

### 2.3. Optimization of the Concentration of Janus Nano-Graphite Temperature-Resistant Foam Stabilizer

**Figure 3:**
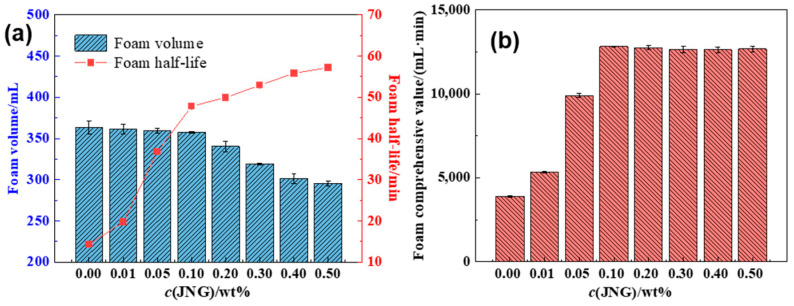
(**a**) Foam volume and foam half-time with different JNG concentrations. (**b**) Foam comprehensive value with different JNG concentrations.

As the concentration of the foam stabilizer increases, the foam volume shows a slight reduction, while the foam half-life significantly increases, both exhibiting an initial rapid increase followed by a more gradual rise (Figure 3).

On one hand, AOS and OHSB molecules are adsorbed in large quantities on the surface of the nanomaterials. As the stabilizer concentration increases, the number of foaming agent molecules adsorbed at the gas–liquid interface decreases. Meanwhile, the surface activity of JNG is enhanced due to the adsorption of foaming agent molecules, leading to an increased likelihood of nanomaterials being adsorbed onto the gas–liquid interface. This further reduces the number of foaming agent molecules adsorbed at the interface, resulting in a decreasing trend in foam volume. On the other hand, as the stabilizer concentration increases, the number of nanomaterials irreversibly adsorbed at the interface rises, leading to a tighter molecular arrangement and an increase in the strength of the gas–liquid interface film. Simultaneously, the foam film thickness increases, thereby enhancing foam stability, as indicated by an increase in foam half-life. Based on the maximum foam performance index, and considering both system cost and improvements in foam performance, the optimal concentration of Janus nano-graphite (JNG) is determined to be 0.10 wt%. Combining these findings with the previously optimized temperature-resistant foaming agent results, the final formulation for the Janus nano-graphite-enhanced foam system is 0.09 wt% AOS + 0.21 wt% OHSB + 0.10 wt% JNG.

### 2.4. Microstructural Changes in JNG-Enhanced Foam

**Figure 4:**
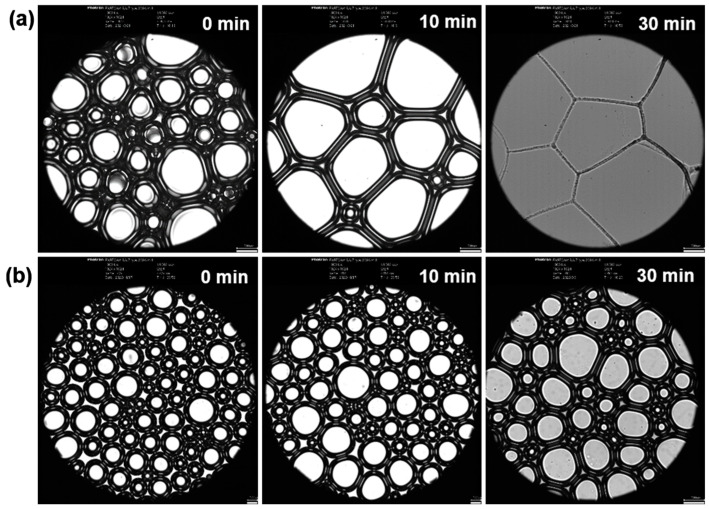
Change of micro morphology of different foam with time. (**a**) Conventional foam (without JNG). (**b**) Enhanced foam (with JNG).

The variation of foam microstructural parameters, such as bubble shape, bubble size, and liquid film thickness, over time provides a clear reflection of foam stability. The more stable the foam, the closer the bubble shapes are to the spherical, the smaller and more uniform the bubble sizes, and the thicker the liquid film. Using an inverted microscope, we observed the microstructures of conventional foam (without JNG) and enhanced gel foam (with JNG). As shown in Figure 4, over time, gas diffusion between adjacent bubbles leads to gradual bubble coalescence, increasing bubble size. However, JNG can adsorb at the gas–liquid interface, increasing the liquid film thickness and slowing the diffusion rate of smaller bubbles into larger ones, thereby reducing the rate of bubble size growth.

**Figure 5:**
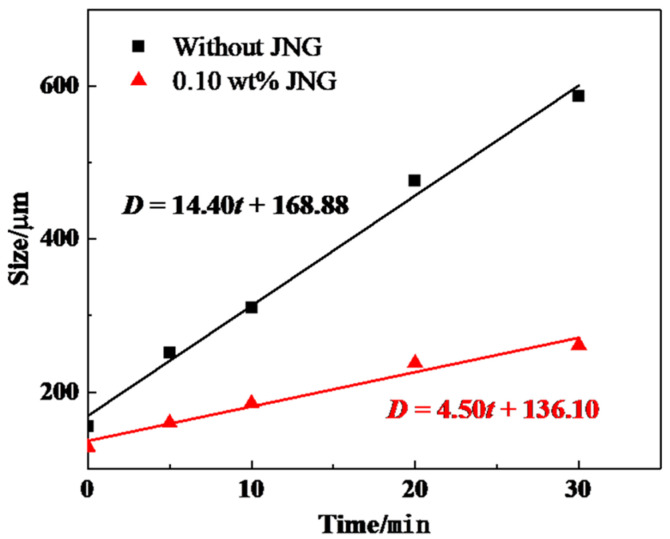
Fitting curve of average bubble size with time in different foam.

Additionally, we utilized the Image J software to measure bubble size distribution at different time points. As shown in Figure 5, the average bubble size for both types of foam exhibited a linear increase over the observation period, and the fitted general equation is presented in Equation (1):

(1) D=a⋅t+b

where *D* is the average bubble size (μm), *t* is the observation time (min), *a* is the bubble growth rate (μm·min^−1^), and *b* is the initial average bubble size (μm).

The smaller the a-value, the slower the bubble growth rate, indicating a more gradual bubble coalescence process. A smaller b-value represents a smaller initial bubble size. According to the fitting results, the enhanced foam exhibited lower values for both a and b, which were 4.50 and 136.10, respectively. This indicates that JNG irreversibly adsorbs at the gas–liquid interface, which, on the one hand, hinders gas diffusion between adjacent bubbles, and, on the other hand, delays liquid drainage from the film, effectively reducing the bubble coalescence rate.

### 2.5. Effect of Janus Nano-Graphite on the Rheological Properties of Foam

**Figure 6:**
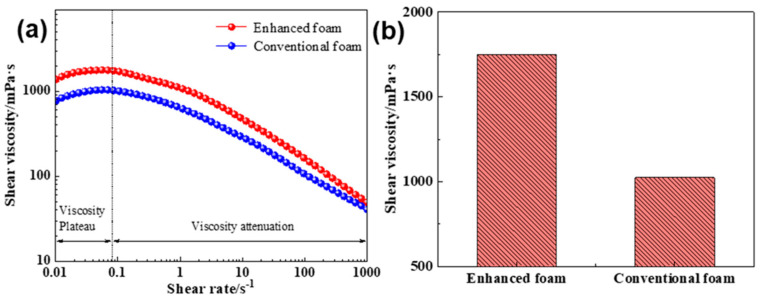
(**a**) The shear viscosity of foams changes with the shear rate. (**b**) Comparison of different foams’ shear viscosity platform values.

The flow behavior and stability of foam fluids in reservoirs are closely related to their rheological properties, particularly shear viscosity, which significantly impacts the effectiveness of foam in controlling gas channeling in heavy oil reservoirs. To analyze the changes in foam rheological properties induced by the addition of Janus nano-graphite, we measured the shear viscosity of different foam systems using a rotational rheometer. As shown in Figure 6, the results indicate that, similar to conventional foam, the enhanced gel foam exhibits non-Newtonian fluid characteristics.

At low shear rates (0.01–0.10 s^−1^), the enhanced foam reaches a viscosity plateau of 1730.2 mPa·s, which is 1.8 times that of conventional foam. As the shear rate increases, the foam viscosity begins to decrease. At a shear rate of 1000 s^−1^, due to the strong shear forces, the viscosity of the enhanced foam decreases to 49.5 mPa·s, though it remains higher than that of the conventional foam. Throughout the test range, the shear viscosity of the enhanced foam was 1.2 to 1.8 times greater than that of conventional foam. This is attributed to the formation of a multi-dimensional network structure within the liquid phase, due to the presence of an appropriate amount of Janus nano-graphite, which increases the internal friction in the continuous liquid phase and results in higher shear viscosity for the enhanced foam.

### 2.6. Evaluation of the Sealing and Channeling Control Performance of JNG-Enhanced Foam in Heterogeneous Reservoirs

**Figure 7:**
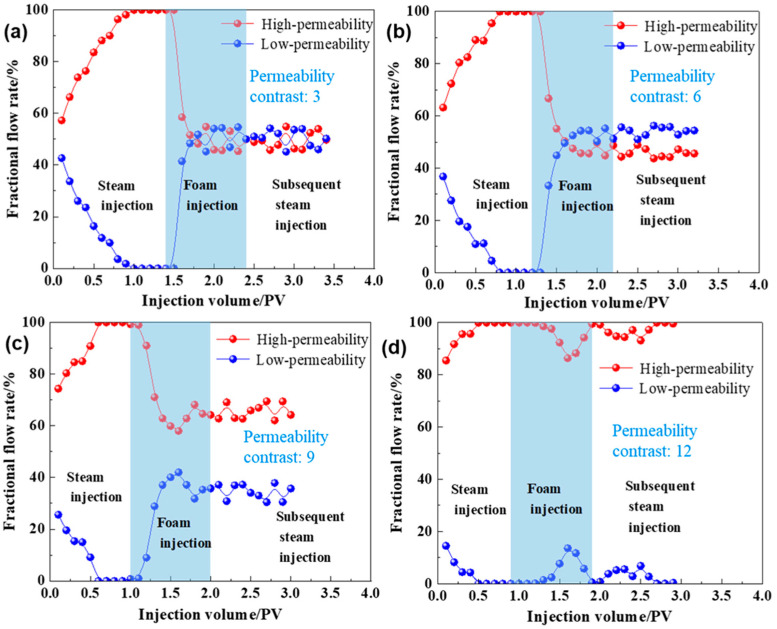
Variation of shunt rate of injection-reinforced foam in reservoirs with different permeability contrast levels. (**a**) 3. (**b**) 6. (**c**) 9. (**d**) 12.

In practical heavy oil reservoirs, significant interlayer heterogeneity often exacerbates steam channeling. The variation in heterogeneity can also impact the effectiveness of foam in controlling steam channeling. As shown in Figure 7, during the steam injection phase, the fractional flow rate in the high-permeability layer is significantly higher than that in the low-permeability layer across different permeability contrasts. The difference in fractional flow rates increases as the permeability contrast becomes larger, indicating that steam channeling occurs in the high-permeability layer. Before foam injection, due to significant differences in pore structure development between high-permeability and low-permeability layers, the high-permeability layer is dominated by large pore channels and throats, resulting in less seepage resistance to steam flow. With continuous injection of high-temperature steam, the high-permeability layer is more prone to forming preferential channeling pathways. Consequently, there is a notable difference in flow diversion rates between the high- and low-permeability layers, necessitating subsequent injection of a channeling sealing system to adjust the steam profile. During the foam injection phase, the enhanced foam preferentially enters the high-permeability layer, where flow resistance is lower. As the foam propagates, the Jamin effect significantly increases the flow resistance in the high-permeability layer, forcing subsequent steam injections to divert towards the low-permeability layer, thus improving steam sweep efficiency. This reduces the fractional flow rate difference between the high- and low-permeability layers, and in some cases, even leads to the fractional flow rate of the low-permeability layer exceeding that of the high-permeability layer—a phenomenon known as “profile inversion.”

When the permeability contrast is between 3 and 9, the enhanced foam effectively improves the steam absorption profile in heterogeneous reservoirs, and the improvement lasts for a considerable time. In particular, at a permeability contrast of 6 (Figure 7b), the profile improvement effect is optimal, with the fractional flow rate of the high-permeability layer decreasing to 43.7%. However, when the permeability contrast reaches 12 (Figure 7d), the injected foam struggles to exert flow control, showing minimal impact on the fractional flow rates of both high- and low-permeability layers, resulting in little to no profile improvement. Based on the fractional flow rate differences between high- and low-permeability layers, enhanced foam is suitable for reservoirs with a permeability contrast of 3 to 9. When the permeability contrast is too large (≥12), the profile improvement effect of the foam is suboptimal.

## 3. Conclusions

A Janus nano-graphite (JNG)-enhanced foam gel system is developed for steam-channeling control in heavy oil reservoirs, using JNG as a high-temperature foam stabilizer and a combination of anionic surfactant AOS and zwitterionic surfactant OHSB as the composite temperature-resistant foaming agent. Under conditions of 250 °C and 5 MPa, the foam half-life reaches 47.8 min, which is 3.4 times that of conventional foam. In the composite system, AOS and OHSB molecules exhibit mutual attraction, with the most significant synergistic effect observed when the concentration ratio c(AOS):c(OHSB) = 3:7. JNG adsorbs at the gas–liquid interface, increasing the thickness of the liquid film and slowing the diffusion rate of small bubbles into larger ones, thus reducing the rate of bubble size growth and enhancing the foam’s thermal stability. The enhanced foam exhibits non-Newtonian fluid behavior, with shear viscosity decreasing as the shear rate increases. The presence of JNG forms a multi-dimensional network structure within the liquid phase, increasing internal friction, resulting in a shear viscosity that is 1.2 to 1.8 times higher than that of conventional foam. The enhanced foam preferentially enters high-permeability layers, blocking steam channeling. The cumulative Jamin effect increases the flow resistance in the channeling pathway, forcing subsequent steam to divert toward low-permeability layers, providing effective control of steam channeling in heterogeneous reservoirs with permeability contrasts ranging from 3 to 9.

## 4. Materials and Methods

### 4.1. Materials

The target reservoir for this study is a heavy oil block in the Shengli Oilfield, where a steam injection pilot test was conducted. The reservoir temperature ranges from 65 °C to 72 °C, the pressure from 3.4 to 6.1 MPa, steam temperature from 220 °C to 300 °C, and formation water salinity from 8412.2 to 37,546.3 mg/L, characterized by a CaCl_2_ water type. The foam system used in the experiment is a nitrogen foam system. Based on these conditions, the experimental temperature was set at 250 °C, the pressure at 5 MPa, and nitrogen was used as the foaming gas. The composition of the synthetic water used in the experiment is shown in Table 2.

**Figure 8:**
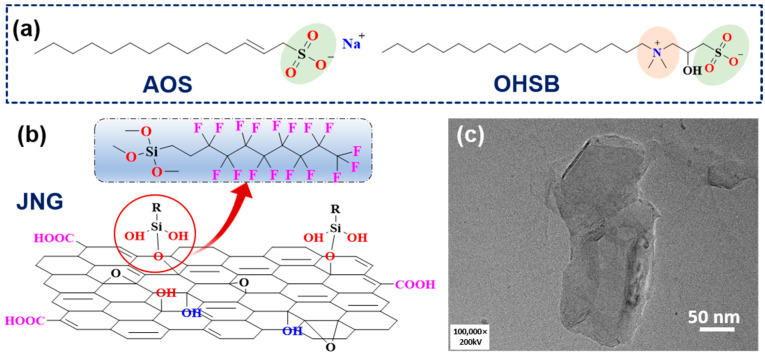
(**a**) The molecular structure of surfactants AOS and OHSB. The molecular structure (**b**) and TEM morphology (**c**) of Janus nanoscale graphite.

The anionic foaming agent was sodium alpha-olefin sulfonate (AOS), and the zwitterionic foaming agent was cetyl hydroxylpropyl sulfobetaine (OHSB). All reagents, including sodium chloride (≥99%), sodium bicarbonate (≥99.5%), calcium chloride (≥96%), magnesium chloride (≥98%), and potassium chloride (≥99.5%), were purchased from Shanghai Aladdin Biochemical Technology Co., Ltd. The temperature-resistant foam stabilizer, Janus nanoscale graphite (hydrophilic on one side, hydrophobic on the other), was synthesized in-lab [49], featuring low surface energy (42.3–47.5 mJ·m^2^), small particle size (182.3 ± 20.0 nm), and low thickness (8.1 ± 0.9 nm). As shown in Figure 8, initially, low-cost natural flake graphite is used as the raw material to produce nano-graphite with abundant surface reactive sites. Subsequently, the nano-graphite is functionally modified using the Pickering emulsion templating method, with 1H,1H,2H,2H-perfluorodecyltrimethoxysilane (PFTS) as the modifier. High-purity nitrogen (99.9%) was supplied by Qingdao Deyi Gas Co., Ltd. (Qingdao, China). The experimental oil was dehydrated and degassed heavy oil from the field, with a viscosity of 904.7 mPa·s at 70 °C. Ultrapure water was laboratory-produced, and quartz sand of various mesh sizes was purchased from Qingdao Kaiwei Powder Engineering Technology Co., Ltd. (Qingdao, China).

### 4.2. Comprehensive Foam Performance Under High Temperature and Pressure

The comprehensive foam performance under high temperature and pressure was evaluated using a high-temperature, high-pressure foam visualization apparatus (TC PMPJ-II Jiangsu Tuochuang Scientific Instruments, Nanjing, China). A 0.5 wt% foaming agent solution was prepared, and 200 mL of the solution was placed in an XGRL-4 high-temperature roller furnace at 250 °C for 24 h of aging, with the roller speed set to 50 rpm. After cooling, the solution was removed for testing. The foam volume and foam half-life of different foaming agents were measured before and after aging at 250 °C and 5 MPa, and a comprehensive foam value was calculated to assess the foam performance under high-temperature conditions. The experimental procedure was as follows:

(a) Nitrogen was introduced into the foam visualization device to fully expel air from the chamber;
(b) 100 mL of the foaming agent solution was pumped into the chamber, and the temperature was raised to the experimental level;
(c) Nitrogen was injected until the experimental pressure was reached. After 10 min of stabilization, the solution was stirred at 5000 rpm for 3 min. The foam volume and foam half-life were recorded.

Foam volume reflects the ease of foam generation, while foam half-life indicates foam stability. The comprehensive foam value was calculated using Equation (2). A higher value indicates a better synergistic effect between foam volume and half-life, and thus, superior foam performance.

(2) $$S=\frac{3}{4}V_{\max}t_{0.5}$$

where *S* is the comprehensive foam value (mL·min), *t*_0.5_ is the foam half-life (min), and *V_max_* is the maximum foam volume (mL).

### 4.3. Intermolecular Interactions of Temperature-Resistant Foaming Agents

The intermolecular interactions of three foaming agent solutions (AOS, OHSB, and AOS/OHSB) were studied using a high-temperature and high-pressure interfacial rheometer (Tracker-H). Surface tension measurements were conducted at 25 °C and 0.1 MPa for different concentrations of the foaming agents, and surface tension versus concentration curves were plotted. Based on surface tension data, the critical micelle concentration (CMC) of each solution was determined. The intermolecular interaction parameters between the two foaming agents were calculated using Equations (3) and (4), which helped assess the strength of synergistic interactions in the mixed system [50].

(3) $$\frac{{\left(x_{1}\right)}^{2}\ln\left(\frac{c_{12}\alpha }{c_{1}x_{1}}\right)}{{\left(1-x_{1}\right)}^{2}\ln\left(\frac{c_{12}(1-\alpha )}{c_{1}\left(1-x_{1}\right)}\right)}=1$$

where *x*_1_ is the mole fraction of AOS in the gas–liquid interface adsorption layer (dimensionless); *c*_12_ is the critical micelle concentration of the mixed foaming agent solution (mol·L^−1^); *c*_1_ and *c*_2_ are the CMCs of AOS and OHSB solutions, respectively (mol·L^−1^); and α is the mole fraction of AOS in the total foaming agent mixture (dimensionless).

(4) $$\beta =\frac{\ln\left(\frac{c_{12}\alpha }{c_{1}x_{1}}\right)}{{\left(1-x_{1}\right)}^{2}}$$

where *β* is the interaction parameter between AOS and OHSB molecules in the mixed micelles (dimensionless).

### 4.4. Microstructural Characterization of Foam

The microstructure of different foam systems over time was observed using an inverted microscope (DMi8, Leica Microsystems, Wetzlar, Germany) at 25 °C and 0.1 MPa. The effect of Janus nanoscale graphite on bubble shape, bubble size, and liquid film thickness, as well as other foam structural characteristics, was analyzed.

### 4.5. Foam Preparation and Rheological Properties

Foam preparation: The conventional foam composition was 0.09 wt% AOS + 0.21 wt% OHSB, while the enhanced foam composition included 0.09 wt% AOS + 0.21 wt% OHSB + 0.10 wt% Janus nanosheets (JNG). A 100 mL foaming agent solution or foaming agent with JNG mixed solution was placed in an LB10G laboratory mixer (Waring, LA, USA), and nitrogen was continuously introduced during high-speed stirring to generate foam.

Rheological properties: After sufficient foaming, a sample was taken and placed in the co-axial cylinder of a rotational rheometer (HAAKE MARS 60, Thermo Fisher Scientific, Dartmouth, CA, USA). The shear viscosity of both the enhanced and conventional foams was measured at 25 °C and 0.1 MPa under varying shear rates.

### 4.6. Enhanced Foam Plugging Performance in Heterogeneous Reservoirs

**Figure 9:**
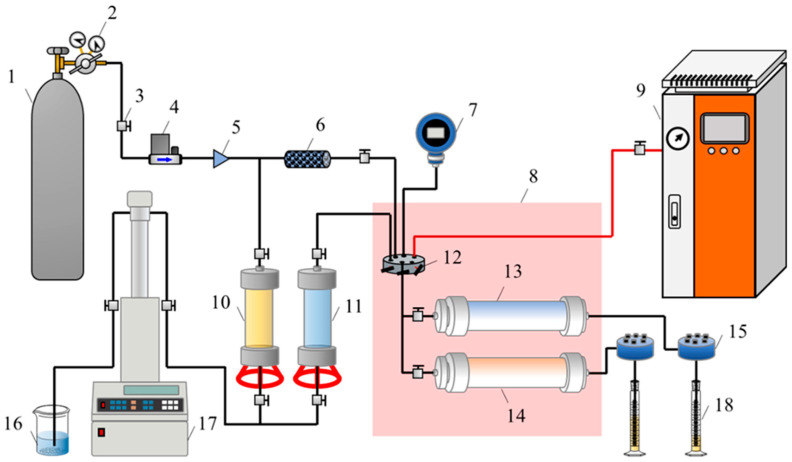
Schematic diagram of high-temperature and high-pressure core flow experiment (double sand packs in parallel). 1—nitrogen cylinder, 2—pressure regulator, 3—valve, 4—gas mass flow controller, 5—check valve, 6—foam generator, 7—pressure gauge, 8—high-temperature oven, 9—steam generator, 10—intermediate container (heavy oil), 11—intermediate container (foaming solution), 12—six-way valve, 13—sand-packed tube (#1), 14—sand-packed tube (#2), 15—back-pressure valve, 16—beaker (ultrapure water), 17—ISCO pump, 18—graduated cylinder.

The effect of reservoir heterogeneity (corresponding to different permeability gradients) on the enhanced foam plugging performance was studied using the flow rate ratio of high-permeability and low-permeability layers as an evaluation index. The experimental procedure is as follows:

(a) A dual-tube parallel high-temperature sand-pack model was prepared using quartz sand by the wet packing method. The permeability of the low-permeability sand-packed model was fixed at 500 × 10^−3^ μm^2^, while the permeability of the high-permeability sand-packed tubes was set at 1500 × 10^−3^ μm^2^, 3000 × 10^−3^ μm^2^, 4500 × 10^−3^ μm^2^, and 6000 × 10^−3^ μm^2^, corresponding to permeability differentials of 3, 6, 9, and 12, respectively. The single model had a diameter of 2.5 cm and a length of 30 cm. The model was saturated with simulated formation water, and the pore volume (PV) was calculated.
(b) The high-temperature, high-pressure core flooding experimental setup was connected according to Figure 9.
(c) The experiment was conducted at 250 °C and 5 MPa back pressure. High-temperature steam was injected into the parallel sand-pack model at a rate of 2.0 mL·min^−1^ until the fluid flow rate at the outlet stabilized. The steam temperature was set to 250 °C with 85% dryness.
(d) Steam injection was stopped, and foam was injected into the parallel sand-pack model at the optimized injection rate and gas–liquid ratio for 1 PV.
(e) After foam injection, steam was reintroduced at 2.0 mL·min^−1^ until the fluid flow rate at the outlet stabilized. The fluid flow rate at the outlet of the high-permeability and low-permeability layers was recorded in real time throughout the experiment. The flow rate ratio was calculated using Equations (5) and (6).

(5) $$DR_{h}=\frac{Q_{h}}{Q}\times 100\%$$

(6) $$DR_{l}=\frac{Q_{l}}{Q}\times 100\%$$

where *DR_h_* and *DR*_1_ are the flow rate ratios of the high-permeability and low-permeability layers, respectively (%); *Q_h_* and *Q*_1_ are the outlet flow rates from the high-permeability and low-permeability layers (mL·min^−1^); and *Q* is the total injected fluid flow rate (mL·min^−1^).

## Figures and Tables

**Table 1:** Interaction parameters between AOS and OHSB molecules.

α	*c*_12_/(mol∙L^−1^)	*c*_1_/(mol∙L^−1^)	*c*_2_/(mol∙L^−1^)	*x*_1_	*β*	ln(*c*_1_/*c*_2_)	pH
0.330	0.005	0.009	0.010	0.425	−2.748	−0.130	6.9

**Table 2:** Simulation of ion composition in formation water.

Ionic Types	Na^+^	K^+^	Ca^2+^	Mg^2+^	HCO^3−^	Cl^−^
Concentration/mg·L^−1^	2632.5	1300.6	260.1	104.0	138.0	5922.3

## Data Availability

The original contributions presented in the study are included in the article, further inquiries can be directed to the corresponding author.

## References

[1] Wang L., Guo J., Li C., Xiong R., Chen X., Zhang X. (2024). Advancements and future prospects in in-situ catalytic technology for heavy oil reservoirs in China: A review. Fuel.

[2] Seng L.Y., Hascakir B. (2021). Role of Intermolecular Forces on Surfactant-Steam Performance into Heavy Oil Reservoirs. SPE J..

[3] Bin L. Research on Steam Flooding Technology in Deep Heavy Oil Reservoir. Proceedings of the 6th International Conference on Mechatronics, Materials, Biotechnology and Environment (ICMMBE 2016).

[4] Head I.M., Jones D.M., Larter S.R. (2003). Biological activity in the deep subsurface and the origin of heavy oil. Nature.

[5] Zhang J. (2023). Performance of high temperature steam injection in horizontal wells of heavy oil reservoirs. Energy.

[6] de Oliveira G.V.B., Coutinho de Araújo J.D., de Meneses Lourenço M.C., Teixeira Araújo de Freitas A.P., Rodrigues M.A.F., de Castro Dantas T.N., Wanderley Neto A.d.O., Alberton Haas D. (2023). Study on Steam and Microemulsion Alternate Injection for Enhanced Productivity in Heavy Oil Fields. Energy Fuels.

[7] Jamshid-nezhad M. (2022). Steam alternating non-condensable gas injection for more heavy oil recovery. Energy.

[8] Cao Y., Liu D., Zhang Z., Wang S., Wang Q., Xia D. (2012). Steam channeling control in the steam flooding of super heavy oil reservoirs, Shengli Oilfield. Pet. Explor. Dev..

[9] Xi C., Qi Z., Zhang Y., Liu T., Shen D., Mu H., Dong H., Li X., Jiang Y., Wang H. (2019). CO_2_ assisted steam flooding in late steam flooding in heavy oil reservoirs. Pet. Explor. Dev..

[10] Zheng W., Fan T., Tan X., Jiang W., Wang T., Xie H. (2021). Numerical Simulations of Chemical-Assisted Steam Flooding in Offshore Heavy Oil Reservoirs after Water Flooding. Geofluids.

[11] Liu H., Wang Y., Zheng A., Sun X., Dong X., Li D., Zhang Q. (2020). Experimental investigation on improving steam sweep efficiency by novel particles in heavy oil reservoirs. J. Pet. Sci. Eng..

[12] Li Y., Zhang W., Wu J., Yang Y., Zhang C., Yang H. (2022). A Comprehensive Method for the Optimization of Cement Slurry and to Avoid Air Channeling in High Temperature and High-Pressure Conditions. Fluid Dyn. Mater. Process..

[13] Wang Y., Liu H., Pang Z., Gao M. (2016). Visualization Study on Plugging Characteristics of Temperature-Resistant Gel during Steam Flooding. Energy Fuels.

[14] Zhu D., Bai B., Hou J. (2017). Polymer Gel Systems for Water Management in High-Temperature Petroleum Reservoirs: A Chemical Review. Energy Fuels.

[15] Chen L., Zhang Z., Zeng H., Huang F., Lu X., Sheng W. (2024). Preparation and Performance of High-Temperature-Resistant, Degradable Inorganic Gel for Steam Applications. SPE J..

[16] Shi Y., He H., Li Y., Ding F., Zhou Z., Xiong N. (2024). High-Temperature-Resistant Epoxy Resin Gel Behavior and Profile Control in Heavy Oil Steam Drive. Energies.

[17] Wei J., Zhang D., Yang E., Shen A., Zhou R. (2024). Effect of foaming agent to CO_2_ ratio on heavy oil recovery efficiency during steam stimulation. Geoenergy Sci. Eng..

[18] Min W., Zhang L. (2024). Application of Flue Gas Foam-Assisted Steam Flooding in Complex and Difficult-to-Produce Heavy Oil Reservoirs. ACS Omega.

[19] Pang Z., Lyu X., Zhang F., Wu T., Gao Z., Geng Z., Luo C. (2018). The macroscopic and microscopic analysis on the performance of steam foams during thermal recovery in heavy oil reservoirs. Fuel.

[20] Yekeen N., Manan M.A., Idris A.K., Samin A.M., Risal A.R. (2017). Experimental investigation of minimization in surfactant adsorption and improvement in surfactant-foam stability in presence of silicon dioxide and aluminum oxide nanoparticles. J. Pet. Sci. Eng..

[21] Hunter T.N., Pugh R.J., Franks G.V., Jameson G.J. (2008). The role of particles in stabilising foams and emulsions. Adv. Colloid Interface Sci..

[22] Wang J., Xue G., Tian B., Li S., Chen K., Wang D., Sun Y., Xu H., Petkov J.T., Li Z. (2017). Interaction between Surfactants and SiO_2_ Nanoparticles in Multiphase Foam and Its Plugging Ability. Energy Fuels.

[23] Zhang Q., Wang H., Han H., Zhao X., Li X., Wang Y. (2023). Experimental study on improving salt resistance of dust suppressing foam with polymers. Fuel.

[24] Hao H., Hou J., Zhao F., Huang H., Liu H. (2021). N_2_-foam-assisted CO_2_ huff-n-puff process for enhanced oil recovery in a heterogeneous edge-water reservoir: Experiments and pilot tests. RSC Adv..

[25] Saxena A., Pathak A.K., Ojha K. (2014). Synergistic Effects of Ionic Characteristics of Surfactants on Aqueous Foam Stability, Gel Strength, and Rheology in the Presence of Neutral Polymer. Ind. Eng. Chem. Res..

[26] Sun Y., Jia Z., Yu B., Zhang W., Zhang L., Chen P., Xu L. (2024). Research progress of nanoparticles enhanced carbon dioxide foam stability and assisted carbon dioxide storage: A review. Chem. Eng. J..

[27] Chaudhry A.U., Muneer R., Lashari Z.A., Hashmet M.R., Osei-Bonsu K., Abdala A., Rabbani H.S. (2024). Recent advancements in novel nanoparticles as foam stabilizer: Prospects in EOR and CO_2_ sequestration. J. Mol. Liq..

[28] Lu T., Li Z., Du L. (2024). Silica aerogel nanoparticle-stabilized flue gas foams for simultaneous CO_2_ sequestration and enhanced heavy oil recovery. J. Clean. Prod..

[29] Zhang Y., Liu Q., Ye H., Yang L., Luo D., Peng B. (2021). Nanoparticles as foam stabilizer: Mechanism, control parameters and application in foam flooding for enhanced oil recovery. J. Pet. Sci. Eng..

[30] Zhang Z., Sun L., Huo X., Liu X., Pan X. (2024). Rheological properties and gas channeling plugging ability in CO_2_ flooding of a hydrophobic nanoparticle-enhanced smart gel system constructed with wormlike micelles. Chem. Eng. Res. Des..

[31] Wang P., You Q., Han L., Deng W., Liu Y., Fang J., Gao M., Dai C. (2018). Experimental Study on the Stabilization Mechanisms of CO_2_ Foams by Hydrophilic Silica Nanoparticles. Energy Fuels.

[32] Chen L., Zeng H., Sun Y., Li G., Zhang Z., Qi J., Tang Z., Xu P. (2023). The foam reinforced with Janus amphiphilic graphene oxide to control steam channeling in heavy oil reservoir. Colloids Surf. A Physicochem. Eng. Asp..

[33] Kang W., Jiang H., Yang H., Li Z., Zhou B., He Y., Sarsenbekuly B., Gabdullin M. (2021). Study of nano-SiO_2_ reinforced CO_2_ foam for anti-gas channeling with a high temperature and high salinity reservoir. J. Ind. Eng. Chem..

[34] Zeng X., Lan X., Zhu H., Liu H., Umar H.A., Xie Y., Long G., Ma C. (2020). A Review on Bubble Stability in Fresh Concrete: Mechanisms and Main Factors. Materials.

[35] Hurtado Y., Franco C.A., Riazi M., Cortés F.B. (2020). Improving the stability of nitrogen foams using silica nanoparticles coated with polyethylene glycol. J. Mol. Liq..

[36] Fujii S., Yokoyama Y., Nakayama S., Ito M., Yusa S.-i., Nakamura Y. (2018). Gas Bubbles Stabilized by Janus Particles with Varying Hydrophilic–Hydrophobic Surface Characteristics. Langmuir.

[37] Correia E.L., Brown N., Razavi S. (2021). Janus Particles at Fluid Interfaces: Stability and Interfacial Rheology. Nanomaterials.

[38] Tohidi Z., Teimouri A., Jafari A., Gharibshahi R., Omidkhah M.R. (2022). Application of Janus nanoparticles in enhanced oil recovery processes: Current status and future opportunities. J. Pet. Sci. Eng..

[39] Jia H., Dai J., Miao L., Wei X., Tang H., Huang P., Jia H., He J., Lv K., Liu D. (2021). Potential application of novel amphiphilic Janus-SiO2 nanoparticles stabilized O/W/O emulsion for enhanced oil recovery. Colloids Surf. A Physicochem. Eng. Asp..

[40] Chen X., Zhang X., Zhang L., Gao Y., Wang C., Hong W., Zhao G., Li L., Liu R., Wang C. (2021). Amphiphilic Janus nanoparticles for imaging-guided synergistic chemo-photothermal hepatocellular carcinoma therapy in the second near-infrared window. Nanoscale.

[41] Tang S., Sun Z., Dong Y., Zhu Y., Hu H., Wang R., Liao H., Dai Q. (2024). Preparation of Amphiphilic Janus-SiO2 Nanoparticles and Evaluation of the Oil Displacement Effect. ACS Omega.

[42] Liang F., Zhang C., Yang Z. (2014). Rational Design and Synthesis of Janus Composites. Adv. Mater..

[43] Wu H.-R., Li G.-L., Xu G.-R., Chang J.-W., Hou K.-P., Shao W.-H., Hou J.-R. (2024). Emulsion properties and plugging performances of active crude oil enhanced by amphiphilic Janus nanosheets. Pet. Sci..

[44] Kulkarni M.M., Bandyopadhyaya R., Sharma A. (2008). Janus silica film with hydrophobic and hydrophilic surfaces grown at an oil–water interface. J. Mater. Chem..

[45] Binks B.P., Horozov T.S. (2005). Aqueous Foams Stabilized Solely by Silica Nanoparticles. Angew. Chem. Int. Ed..

[46] Duan Y., Zhao X., Sun M., Hao H. (2021). Research Advances in the Synthesis, Application, Assembly, and Calculation of Janus Materials. Ind. Eng. Chem. Res..

[47] Borówko M., Rżysko W., Słyk E. (2019). Self-assembly in two-dimensional mixtures of Janus disks and isotropic particles. J. Chem. Phys..

[48] Luo D., Wang F., Zhu J., Tang L., Zhu Z., Bao J., Willson R.C., Yang Z., Ren Z. (2017). Secondary Oil Recovery Using Graphene-Based Amphiphilic Janus Nanosheet Fluid at an Ultralow Concentration. Ind. Eng. Chem. Res..

[49] Sun N., Yao X., Xu Z., Li J., Yang N., Lyu D., Zhao G., Dai C. (2023). Janus nanographene oxide with aerophilic/hydrophilic characteristics for enhancing foam stability in high-temperature reservoirs. J. Mol. Liq..

[50] Syed A.H., Yekeen N., Padmanabhan E., Idris A.K., Mohshim D.F. (2020). Characterization of lauryl betaine foam in the Hele-Shaw cell at high foam qualities (80%–98%). Pet. Sci..

